# Case Report: Expanding Clinical, Immunological and Genetic Findings in Sideroblastic Anemia With Immunodeficiency, Fevers and Development Delay (SIFD) Syndrome

**DOI:** 10.3389/fimmu.2021.586320

**Published:** 2021-04-14

**Authors:** Leonardo Oliveira Mendonca, Alex Isidoro Prado, Izelda Maria Carvalho Costa, Marcia Bandeira, Rafael Dyer, Samar Freschi Barros, Karen Francine Khöler, Luiz Augusto Marcondes Fonseca, Jorge Kalil, Fabio Morato Castro, Myrthes Anna Maragna Toledo-Barros

**Affiliations:** ^1^ Clinical Immunology and Allergy, School of Medicine, University of São Paulo, São Paulo, Brazil; ^2^ Laboratory for Immunological Investigation (LIM-19), Heart Institute, University of São Paulo, São Paulo, Brazil; ^3^ Discipline of Dermatology, University of Brasília, Brasília, Brazil; ^4^ Department of Pediatric Rheumatology, Hospital Pequeno Príncipe, Paraná, Brazil; ^5^ Department of Surgical Pathology, School of Medicine, University of São Paulo, São Paulo, Brazil

**Keywords:** SIFD, recurrent fever, erythema nodosum, B-cell deficiency, TRNT1

## Abstract

Since the first description of the syndrome of sideroblastic anemia with immunodeficiency, fevers and development delay (SIFD), clinical pictures lacking both neurological and hematological manifestations have been reported. Moreover, prominent skin involvement, such as with relapsing erythema nodosum, is not a common finding. Up to this moment, no genotype and phenotype correlation could be done, but mild phenotypes seem to be located in the N or C part. B-cell deficiency is a hallmark of SIFD syndrome, and multiple others immunological defects have been reported, but not high levels of double negative T cells. Here we report a Brazilian patient with a novel phenotype of SFID syndrome, carrying multiple immune defects and harboring a novel mutation on TRNT1 gene.

## Introduction

A syndrome characterized by sideroblastic anemia, with associated B-cell immunodeficiency, periodic fevers and development delay (SIFD), was first described as an isolated entity in 2013, followed by the molecular identification of the causative gene, TRNT1, by the same group in 2014 (1, 2). Initially, diagnostic criteria specified the presence of clinical signs referred to in the acronym associated with the syndrome, yet the first report also highlighted several other observable clinical signs. Over time, pleiotropic clinical manifestations were observed, notably in the absence of sideroblastic anemia, developmental delay and a broad spectrum of immunological defects. Moreover, skin manifestations were not commonly observed in this disorder (3, 4).

The gene encoding transfer RNA (tRNA) nucleotidyl transferase 1 (TRNT1) is responsible for the formation of an enzyme essential to the synthesis of the 3′-terminal CCA sequence in tRNA molecules in the nucleus and mitochondria. Mutations in gene TRNT1 result in partial loss-of-function defects, leading to metabolic abnormalities in both the mitochondria and cytosol that account for the multiple phenotypes thus far reported (5). At the time of this publication, no genotype and phenotype correlations had been reported.

Here we report a novel SIFD phenotype characterized by multiple immunological defects in a Brazilian patient harboring a novel bi-allelic mutation in gene TRNT1.

## Patient and Method

### Clinical Data and Genomic Sequencing

Clinical data were retrieved from the patient’s records after her parents provided written consent for the publication of any potentially identifiable images or data included herein. Genomic DNA was extracted from blood samples using a QIAamp^®^ DNA Blood Maxi Kit (Qiagen^®^, Valencia, CA, USA). PBMCs were obtained by density gradient centrifugation  (d=1.077 g/ml). Primers directly targeting exon 4 of the *TRNT1* gene were designed. Sanger sequencing was performed for genetic confirmation and familial segregation in accordance with standard procedures.

### Quantification of B and T Lymphocyte Phenotypes by Flow Cytometry

Peripheral blood mononuclear cells (PBMC) were stained with titrated mouse anti-human monoclonal antibodies (mAbs) (all from BD Biosciences). Fluorescence minus one (FMO) control was set up for CD45RA marker.

Flow cytometry was performed in FACSCanto II (BD Biosciences) and the analyses were made in FlowJo 9.9.5 software (TreeStar Inc, San Carlos, CA, USA). After exclusion of cell doublets, sequential gating of PBMC was performed in the lymphocyte region. For T lymphocytes, after gate of CD3+ T cells, followed by discrimination of CD8+ and CD4+ markers, we analyzed the T lymphocyte naïve/memory subpopulations through Boolean gates: T naive (CD45RA^+^CCR7^+^CD27^+^), T central memory (TCM) (CD45RA^-^CCR7^+^CD27^+^), T effector memory (TEM) (CD45RA^-^CCR7^-^CD27^+^) and T effector memory with RA re-expression (TEMRA) (CD45RA^+^CCR7^-^CD27^-^). To analyze the phenotypes CD3^+^ TCRaß CD4^-^ CD8^-^, CD3^+^ HLA-DR^+^ and CD3^+^ B220^+^, the cells were gated on CD3^+^ region, after exclusion of doublets and death cells. The phenotype CD27^+^ was analyzed in B lymphocyte region (CD20^+^ cells).

Lymphocyte subsets absolute counts were calculated using the percentages obtained in flow cytometry. The subset percentages analyzed were referred to total lymphocyte counts for T and B cells.

## Results

### Clinical Case

**Figure 1:**
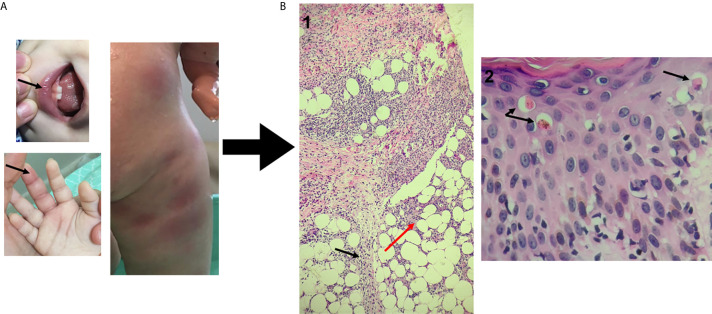
Dermatological and histopathological findings. **(A)** - Oral ulceration and dactylitis (black arrows) and skin rash observed during fever flares resembling erythema nodosum. **(B)** Hematoxylin-eosin staining of skin biopsy (punch) previously fixed in 10% neutral buffered formalin. 1 - (100x magnification): Septal panniculitis (black arrow) with foci of inflammatory cells extending into adjacent fat lobule (red arrow). 2 - (400x magnification): epidermal spongiosis and lymphocyte exocytosis with dyskeratotic cells (arrow) and interface dermatitis with vacuolar changes in the basal layer.

**Figure 2:**
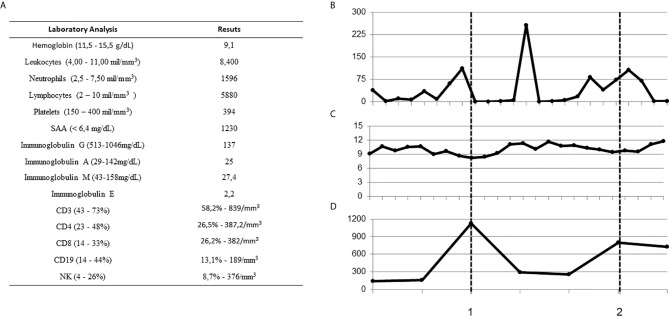
Baseline laboratory findings and treatment response over time. **(A)** Laboratory findings at baseline (normal ranges in brackets) **(B–D)** CRP, hemoglobin and Immunoglobulin G over time during febrile episodes and at basal levels. Dashed lines indicate immunoglobulin replacement, 1: single shot, and 2: after monthly infusions. (CRP, C-reactive protein; SAA, serum amyloid A).

The patient, a 3-year-old Brazilian female, was born to non-consanguineous healthy parents and experienced recurrent episodes of fever since the first month of life. Initially, febrile episodes were characterized by high fever lasting five days and associated with arthritis and dactylitis. In her ninth month of life, the appearance of a diffuse painful skin eruption was noted, marked by nodules and plaques, erythematoedematous rash and infiltrate, resembling erythema nodosum (**Figure 1**). The child was admitted on an almost monthly basis; on some occasions her symptoms were attributed to defined infections (mono-like), yet in other instances infections were undefined–these were somewhat responsive to systemic antibiotics. In her eleventh month of life, during another episode of fever, low levels of immunoglobulin G prompted the initiation of intravenous immunoglobulin (IVIG) replacement. IVIG had an effect on infectious episodes and consequently reduced the frequency of hospital admissions, but skin rash and arthritis remained uncontrolled. A biopsied skin specimen from her left leg revealed septal panniculitis, thus confirming the clinical suspicion of erythema nodosum (**Figure 1**). At the age of two years, while high doses of steroids improved skin and osteoarticular symptoms, the patient failed to respond to several steroid sparing agents. Due to severe side effects, steroid administration was suspended. Monthly doses of IVIG (500 mg/kg) associated with anti-TNF alfa (etanercept) at the dose of 25 mg subcutaneously every week led to the control of fever, skin and osteoarticular symptoms. While anemia was a constant finding, sideroblastic changes were not evidenced in peripheral smears. In addition, no clinical signs of neurological development impairment were seen. All relevant laboratory analyses are summarized in **Figure 2**.

### Genetic Analysis

**Figure 3:**
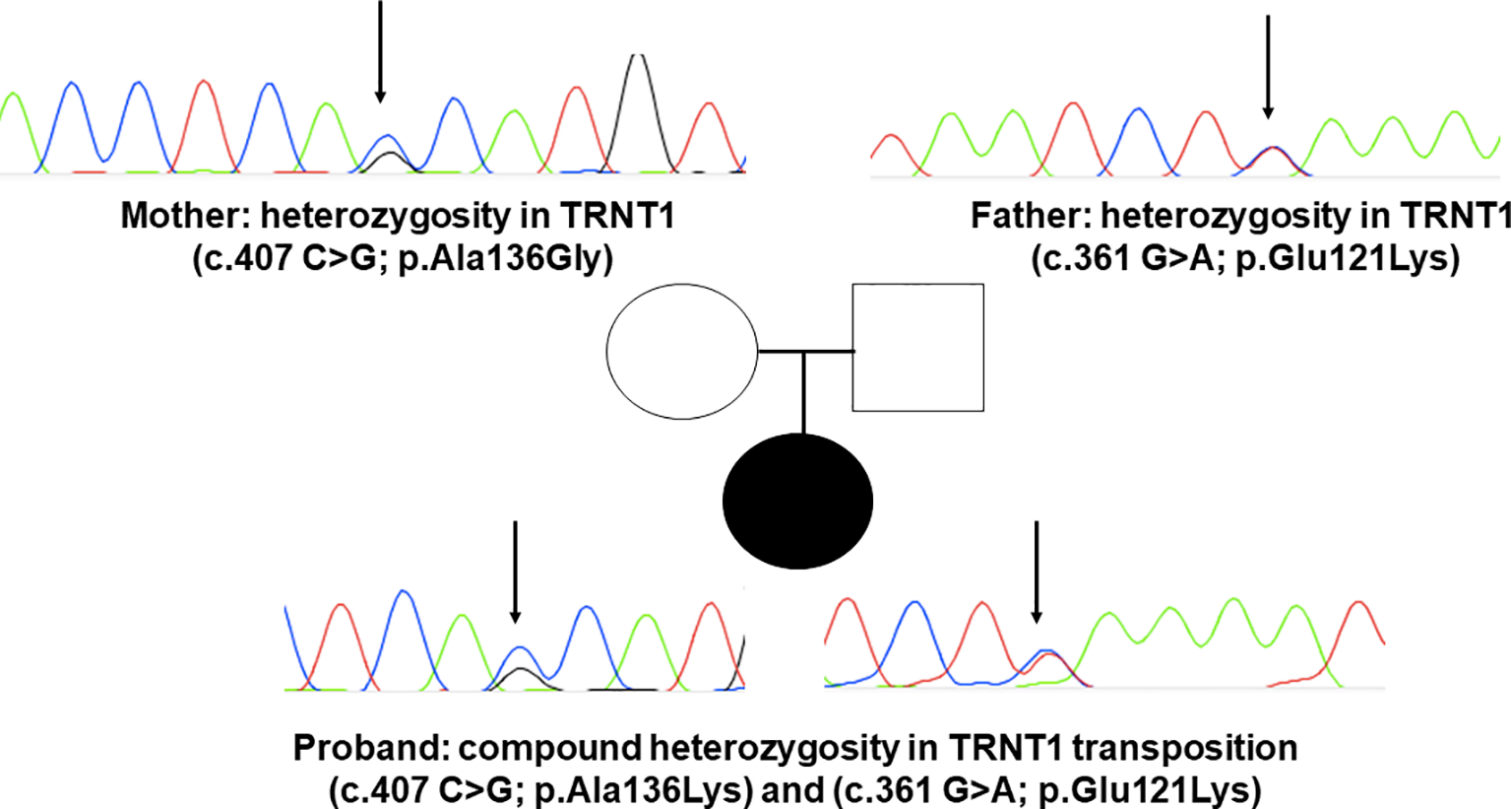
DNA sequence electropherograms demonstrating mutations p.Ala136Lys and p.Gly121Lys in *TRNT1*. Sanger sequencing results for TRNT1 mutations in SFID in compound heterozygous status in the proband. The father carries p.Gly121Lys and the mother p.Ala136Lys, both in heterozygous fashion. Electropherograms detail each of the two newly reported mutations, indicated by black narrows.

Upon suspicion of an inborn error of immunity (IEI), commercially available whole exome sequencing was solicited, revealing two novel mutations in exon 4 of TRTN1 (c.361 G>A; p.Glu121Lys and c.407 C>G;p.Ala136Gly). Neither of these mutations had been previously reported in SIFD patients. Subsequently, both variants were confirmed and segregated by Sanger sequencing, confirming inherited transposition of the variants (**Figure 3**).

### Immunological Analysis

Consistently with expected findings in SIFD patients, very low frequencies of CD19 (B lymphocytes), low B naive cells, normal B memory cells and low levels of immunoglobulins G, M and A were found. The patient also presented positive IgG antibodies for herpes simplex virus and anti-VCA. Levels of dense fine speckled nuclear ANA fluctuated, with titers ranging from 1:160 to zero. Anti-ENA, as well as C3 and C4 complement titers, were within normal ranges (**Table 1** and **Figure 2A**).

**Table 1:** Peripheral lymphocyte repertoire evidencing multiple immune defects.

Lymhocyte	Total number	%	Age Reference (3y)
CD3+	14936	**76**	66.2 (57.1-72.7)
CD4+	7493	**50,2**	37.7 (27.7-46.3)
CD8+	5068	**33,9**	21.9 (15.7-33.8)
CD19+	139	**6,12**	19.3 (13.3-26.7)
CD4+ naïve	1476	70,8	70.30 (46.14-84.40)
CD4+ TCM	247	**11,8**	26.40 (13.88-48.12)
CD4+ TEM	65	**3,12**	2,8 (0.94-6.46)
CD4+ TEMRA	4	0,192	0.2 (0.00-1.36)
CD8+ naïve	503	**59,6**	63.5 (36.80-83.16)
CD8+ TCM	78	**9,24**	15.8 (5.18-31.66)
CD8+ TEM	52	**6,16**	3.40 (0.70-11.22)
CD8+ TEMRA	18	**2,13**	15.5. (0.84-33.02)
CD20+ CD27+ (LB mem)	289	7,24	7.70 (3.60-18.55)
CD20+ CD27- (LB naive)	461	**11,4**	76.20 (59.59-85.28)
CD3+ TCRab CD4- CD8-	390	**6,3**	<1,5%
CD3+ B220+	2266	36,7	-
CD3+ TCRab	4318	92	-
CD3+ TCRgd	610	13	-

In bold are the aberrant expressions when compared to the reference range. As expected in SIFD disease, marked low levels of B (CD19 cells) can be observed and an unusual observation of very high levels of double negative T cells (TCR α/β).

The peripheral T lymphocyte repertoire evidenced multiple other immune defects in T naïve and memory cells, in addition to high frequencies of total T lymphocytes, as well as CD4 and CD8. T CD4 naïve and CD4 TEMRA cell expression were normal, but low frequencies of TCD4 CM and high TCD4 EM were noted. Expression levels of TCD8 naïve, TCM and TCD8 TEMRA were low, while high amounts of TCD8 EM cells were quantified. We additionally detected excessive peripheral expression of double-negative T cells (**Table 1**).

## Discussion

Here we report a case of a SIFD patient lacking signs of sideroblastic anemia and neurodevelopment delay and presenting with a new autoinflammatory phenotype characterized by recurrent episodes of fever and erythema nodosum. Skin manifestations in SIFD syndrome are rare; just one case with ichthyotic skin changes was initially reported (1). A later review of the 17 cases previously reported in the literature did not evidence consistent mucocutaneous manifestations in SIFD. However, one adult patient presented lichen sclerosus et atrophicus and morphea (6). Panniculitis, but not relapsing erythema nodosum, was previously reported in one SIFD patient (7).

Since the first publication on SIFD, several studies have described other patients harboring disease-causing mutations in *TRNT1*, notably bearing phenotypes incompatible with the original description, such as the present case. Since TRNT1 is encoded in the nucleus, as required in both cytoplasm and in mitochondria, specific mutations may impact the ability of TRNT1 to refold properly once transported into mitochondria, thus giving rise to the spectrum of phenotypes observed. Indeed, studies mapping mutations along the TRNT1 gene have suggested that mild phenotypes are located around the N or C terminal domains of this gene, as was seen in our patient (8, 9). We report two novel mutations in the TRNT1 gene and even if functional analysis of the protein expression was not performed, taking into account the clinical and immunological phenotype altogether with the *in-silico analysis* of the mutations found we strongly believe that both are causative of SIFD syndrome.

Multiple immunological defects or phenomena have been described in patients with SIFD. While a significant reduction in B cells is noted, other lymphocyte classes seem to initially remain preserved, but then progressively decline, resulting in profound B, T and NK lymphopenia. One study that performed extensive immunophenotyping revealed increased TCD8 cells, which was also observed in our patient. Curiously, a low TCD8 frequency was found prior to IVIG replacement, which may reflect positive immune modulation by IVIG. Contrary to previous descriptions, we detected normal levels of TCD4 terminally differentiated effector memory helper T lymphocytes (CD4 TEMRA), as well as increased numbers of CD4 effector memory lymphocytes (CD4 EM). Similarly to other studies, we also found a lower percentage of switched memory B cells (1–3). We call attention to a relevant consideration, as no reference range for these cells exists in the Brazilian population; therefore, the range reported in a Chinese study (9) was employed, which must be taken into account when interpreting the present results.

A recent report on a patient carrying a mutation in the N terminal region of *TRNT1* identified high levels of interferon-α (IFN-alpha) and elevated expression of interferon-stimulated genes, and thusly hypothesized that this signature could be relevant in some clinical phenotypes; notably, no skin involvement was described (10). The IFN-alpha signature is a hallmark of proteasome-associated autoinflammatory syndromes (PRAAS) in which skin eruptions, such as panniculitis, as was observed in our patient, are a common finding (11). The peripheral expression of double-negative (DN) T cells, a marker of apoptotic cell death, is a hallmark of Autoimmune Lymphoproliferative Syndrome (ALPS) and other ALPS-related syndromes, including some autoinflammatory disorders (12, 13). The present observation of considerably high numbers of DN T cells has not been previously reported in SIFD patients to date, thus expanding the immunological features of SIFD.

## Conclusion

This present case constitutes the first report of SIFD in Brazil, and serves to enhance the range of clinical, immunological and genetic findings associated with this syndrome. IVIG replacement has appeared to have a positive immunomodulatory effect in affected patients, and *TRNT1* mutations should be considered in patients with ALPS-like syndromes. Autoinflammatory signs, such as recurrent fever and erythema nodosum, should also prompt consideration for *TRNT1* genetic screening. As aberrant TRNT1 functioning seems to stress proteasome activity, further study may shed light on the therapeutical relevance of these cell machinery interactions in both SIFD and PRAAS patients.

## Data Availability Statement

The authors acknowledge that the data presented in this study must be deposited and made publicly available in an acceptable repository, prior to publication. Frontiers cannot accept a article that does not adhere to our open data policies.

## Ethics Statement

The studies involving human participants were reviewed and approved by University of São Paulo—School of Medicine. Written informed consent to participate in this study was provided by the participants’ legal guardian/next of kin.

## Author Contributions

LM, AP, SB, and KK: article draft, sanger sequencing, performance of flow cytometry, and data analysis. IC, MB, and RD: derma-pathological analysis and data interpretation. LF, JK, FC, and MT-B: article review. All authors contributed to the article and approved the submitted version.

## Conflict of Interest

The authors declare that the research was conducted in the absence of any commercial or financial relationships that could be construed as a potential conflict of interest.
